# A Mobile-Based Approach to Enhance Knowledge of Infant and Young Child Feeding Among Teenage Mothers in Nigeria: A Randomized Controlled Trial

**DOI:** 10.3390/nu17030414

**Published:** 2025-01-23

**Authors:** Mercy E. Sosanya, Oluwatosin D. Adesanya, Hauwa E. Rufai, Jeanne H. Freeland-Graves

**Affiliations:** 1 Department of Nutritional Sciences, University of Texas at Austin, Austin, TX 78712, USA; jfg@mail.utexas.edu; 2 Department of Nutrition and Dietetics, The Federal Polytechnic, Bauchi, Bauchi 740102, Nigeria; otochidorothy@fptb.edu.ng (O.D.A.); rufaihauwaenya@gmail.com (H.E.R.)

**Keywords:** childhood malnutrition, infant and young child feeding (IYCF), Nigeria, teenage mothers, BabyThrive app, mobile game, mHealth, serious game, exclusive breastfeeding, complementary feeding, maternal knowledge

## Abstract

**Background/Objectives:** The second highest prevalence of childhood malnutrition in the world is found in Nigeria. Infant and young child feeding practices (IYCF) are crucial predictors of the nutritional status of children. This study evaluated the effects of utilization of the BabyThrive app versus control on IYCF knowledge of Nigerian teenage mothers. **Methods:** A parallel, randomized controlled trial was conducted with 194 low-income teenage mother–child (0–2 years) dyads in Nigeria. Outcome measures included knowledge concerning food type for an infant, exclusive breastfeeding, complementary feeding, and total IYCF knowledge scores. **Results:** No significant differences in demographic characteristics or IYCF knowledge were found at baseline. Post-intervention, almost all BabyThrive participants vs. only 36.1% of controls were aware that expressed breast milk is the ideal food for an infant <6 months, when a mother resumes work (*p* < 0.05). As compared to the BabyThrive group, knowledge of how to maintain breast milk supply (5.2% vs. 83.5%), the duration of safety of breast milk at room temperature (15.4% vs. 85.6%), responsive feeding (58.8% vs. 96.9) and dietary diversity (15.5% vs. 99%) was substantially lower in controls (*p* < 0.05). Mean knowledge on exclusive breastfeeding (25.17 ± 1.37 vs. 19.87 ± 1.80), complementary feeding (15.68 ± 0.60, vs. 13.51 ± 1.21) and total knowledge scores (46.8 ± 1.79 vs. 38.65 ± 2.71) was higher in the BabyThrive group (*p* < 0.05). **Conclusions:** In sum, the BabyThrive app significantly increased maternal IYCF knowledge in comparison with controls. It will be a useful tool to improve maternal IYCF knowledge in resource-limited areas.

## 1. Background

The second highest prevalence of childhood malnutrition in the world is found in Nigeria [1]. Although poverty is endemic in this country, the most important feeding practices necessary for children to survive and thrive are not capital-intensive [2,3]. For instance, exclusive breastfeeding for six months, as recommended by the World Health Organization, is far cheaper and safer than formula feeding in communities with limited or no access to potable water [2,4]

Early marriage is common in the northwestern region of Nigeria, where the median age at marriage is 15.8 years, and up to 57% of females have no education [5,6]. In states like Bauchi in northern Nigeria, the burdens of teenage pregnancies and child undernutrition are exceedingly high, with over 40% of girls 15–19 years bearing children [6]. This situation is not ideal, as teenage mothers may lack the necessary knowledge, skills and self-efficacy required to practice appropriate child feeding [7]. In this area, the period between marriage and first birth is short, and does not provide sufficient time to learn about how to care for an infant [8,9]. In an investigation by the United Nations Children’s Fund (UNICEF) into child survival and nutrition practices in Nigeria, the majority of adolescent girl reported that they had not received any nutritional education, in contrast to large proportions of older women who had received nutritional counseling [1]. Furthermore, adolescent girls have limited access to social services, with large proportions (44.9–53.4%) reporting insufficient economic support from parents, inadequate access to education and other government services, and a suboptimal availability of reproductive health information [10]. Reproductive health education is largely absent from the Nigerian basic education curriculum in secondary schools, except in a few states in the southwest where it is taught [11,12]. Additionally, numerous studies have shown that in comparison to older mothers, Nigerian teenage mothers are significantly less likely to use antenatal and other health services due to limited education, economic constraints, diminished autonomy, ineffectual social support systems, social stigmas associated with teenage childbirth, and condemnatory attitudes of health workers [13,14,15,16].

In northern Nigeria, numerous interventions have attempted to ameliorate child malnutrition and improve IYCF knowledge and practices, but the impact of these programs has been limited [17]. To date, sub-optimal IYCF practices have been documented to remain widespread among mothers in this region [6]. In 2023, only about one-third of Nigerian infants (0–5 months) were exclusively breastfed [1]. Greater health knowledge on the part of parents is a critical determinant of desirable dietary practices, as well as nutritional and overall health behaviors in both parents and children [18,19,20]. Recent cross-sectional studies and nutrition education interventions have established that knowledge of child feeding and/or general nutrition is an important predictor of child feeding intentions/practices and child anthropometric status [21,22]. Thus, urgent and scalable interventions are needed to improve the knowledge of teenage mothers concerning infant and young child feeding and nutrition.

The use of mHealth apps on mobile phones is one possible strategy to improve the knowledge of mothers concerning child feeding [23]. According to the World Health Organization (2011), mHealth can be defined broadly as a branch of eHealth that utilizes mobile technologies. These include mobile phones, patient monitoring devices, personal digital assistants, and other wireless devices to support medical and public health practices [24]. The ubiquitous nature of mobile phones in developing countries creates an excellent and affordable opportunity for knowledge acquisition, since mobile networks in these areas exceed other infrastructure and services (such as paved roads and electricity) [24,25,26]. For instance, in Nigeria’s population of almost 230 million, there were approximately 218 million active mobile phone lines in 2023; about one phone line per individual [27,28]. Additionally, in a study of 726 Nigerian females aged 12–30 years, large proportions of respondents living in four northern states either owned mobile phones or had access to devices belonging to relatives, as follows: Adamawa State (45.6% owned, 54.4% had access through relatives), Gombe (89.5% owned, 2.4% had access), Kaduna (58.6% owned, 41.4% had access) and Taraba (70.1% owned, 29.9 had access) [29]. Consequently, the number of mHealth interventions focusing on reproductive, maternal, newborn, and child health is increasing [30]. Nonetheless, a recent review of mHealth interventions targeting maternal and child healthcare in developing countries documented that 18 of 28 studies focused on maternal health, and 8 focused on child immunization, but only 2 of these studies were aimed at improving either infant and young child feeding or breastfeeding [30].

Substantial improvements in nutrition education are still needed in developing countries. In northern Nigeria, an IYCF program was implemented by a collaboration of state governments and international development partners [17]. It provided individualized counseling in health facilities, volunteer-led community counseling to mothers, and community meetings for fathers and grandmothers [17]. A qualitative evaluation of the program indicated a preference of mothers for individualized counseling, as compared to grandmothers, who preferred support group sessions. Yet, fathers remained largely uninvolved [17]. Problems identified with the projects included the limited financial remuneration of community volunteers, time constraints, and challenges with transportation [17]. The current project is an mHealth intervention that targets a wider client base, with decreased cost, time, space and requirements for personnel training and transportation [31].

Mobile games delivered on a phone can be a vehicle for change due to their capacity to modify knowledge, behaviors and skills pertaining to health, while providing entertaining leisure activity [32,33,34]. Games designed specifically based on pedagogical principles for a defined educational purpose in addition to entertainment are known as serious games [35,36,37]. Serious mobile games are suitable for adolescent health interventions, given their proclivity to play games and utilize technology [38,39]. A recent meta-analysis of 18 papers in developed countries that focused on addressing childhood obesity (5–17 years) concluded that gamification and the utilization of game elements improved the nutritional knowledge and eating habits of children [40]. Similarly, in a meta-analysis including 26 studies in Western countries, serious games were found to increase the nutrition knowledge of children, resulting in healthier diets [34]. Although gamification is popular for exercise/fitness and obesity-focused apps, few apps have focused on changing IYCF constructs. In Indonesian mothers, a puzzle game simulation significantly increased nutritional knowledge and child (0–2 years) feeding practices [41]. However, no studies have applied mobile gaming techniques to facilitate changes in IYCF knowledge in Africa, to the best of the knowledge of the authors. This research proposed to develop an offline gaming app called BabyThrive. It has been validated in Nigerian teenage mothers for improving maternal IYCF knowledge, attitudes and practices [42]. In the present research, the effects of the BabyThrive app versus controls on the knowledge of exclusive breastfeeding and complementary feeding of Nigerian teenage mothers are evaluated.

## 2. Methods

### 2.1. Design of Experiments and Participants

A parallel, randomized controlled trial was conducted over 6 months with 194 low-income mother–child (0–2 years) dyads.

**Figure 1:**
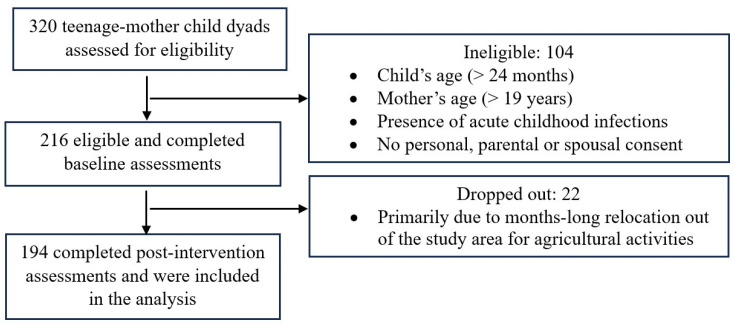
CONSORT flow diagram for a randomized controlled trial of the BabyThrive app.

Participants were recruited from the suburbs of Bauchi, the Bauchi State capital, and Abuja, the Federal Capital Territory, of Nigeria. Mothers (14–19 years) who had access to smartphones, with at least one child (0–2 years) having a negative weight-for-length Z-score (<0), were included in the study. Children with acute infections, metabolic or feeding issues, and mothers younger than 14 years of age or with HIV-positive status were excluded. A power analysis was performed using the G*Power 3.1.2 program (University Kiel, Germany) [43]. Assuming a 25% attrition (including incompletes), the final sample size was 216. Using a 1:1 allocation ratio, mother–child pairs were randomly assigned to either the BabyThrive group or a control group by drawing lots (control group, *n* = 108 and BabyThrive group, *n* = 108). A total of 194 participants who completed the study were included in the final analyses, reflecting an 11.8% attrition rate. The CONSORT flow diagram is presented in Figure 1.

After baseline data collection, mothers in both groups continued to receive all routine primary healthcare services. In the intervention group, research assistants installed the BabyThrive app on the smartphones of participants, taught them how to use the app, and waited for each participant to play the game from the beginning to the end. Similarly, three months into the intervention, the research assistants visited each participant and watched them play the game from the beginning to the end. Participants in the BabyThrive group received monthly phone call reminders to use the app, and challenges faced were addressed during these phone conversations. Throughout the intervention, the app was reinstalled on participants’ phones as required, due to lost/damaged/stolen devices, or other problems (including deletion of the app because of temporary problems with phone storage space). The control group received a delayed intervention (installation of the app after study completion). The risks and benefits of the study were explained to all mothers and their spouses/parents, and all participants and their spouses/parents provided written, informed consent.

### 2.2. The BabyThrive App

The BabyThrive app is a valid, two-dimensional, role play video game that features training on correct breastfeeding and complementary feeding recommendations [33]. It also includes 30 nutritionally adequate recipes from locally available and affordable foods to ensure dietary diversity. The BabyThrive app is available in English and the Hausa language, which is predominantly spoken in northern Nigeria. It is fully functional offline once installed, mitigating the barrier of unreliable and expensive internet connectivity. Detailed descriptions of the BabyThrive app, and the procedures for the development and validation of the app, are documented elsewhere [42]. The gaming app contains information related to food type for an infant. Examples are what to feed infants <6 months, the need for breast milk expression and how to successfully express breast milk in the absence of sophisticated equipment. With respect to exclusive breastfeeding, the app presents information on the need, appropriate duration, and benefits of exclusive breastfeeding to the child, mother and community as a whole. It also provides suggestions on maintaining breast milk supply, breastfeeding techniques and positions, the safety of expressed breast milk without refrigeration, overcoming breastfeeding difficulties, and the concepts of fore and hind milk. The complementary feeding information provided in the app includes the appropriate age of introduction of complementary foods, adequate meal frequency, quantities per meal, consistency, dietary diversity, the provision of eggs or animal flesh, the need for fruit and vegetable consumption, and responsive feeding techniques for an infant rejecting complementary foods.

### 2.3. Outcome Measures

The effects of BabyThrive on maternal IYCF knowledge were determined by participant responses to a validated Teen Moms Child Feeding Questionnaire for Sub-Saharan Africa [44]. This questionnaire was administered at baseline and 6 months to both study groups. Summaries of responses to each knowledge question in the questionnaire were computed. The summation of scores from questions relating to food type for an infant (6 points maximum), exclusive breastfeeding (24 points maximum), complementary feeding (16 points maximum) and total knowledge of child feeding (48 points maximum) was calculated. The efficacy of the app was determined by comparing IYCF knowledge across both study groups.

## 3. Statistical Analyses

Descriptive statistics such as frequencies, percentages, means and standard deviations, and medians were computed for the variables. Categorical variables included sociodemographic variables and proportions of responses to specific knowledge questions on the Teen Moms Child Feeding Questionnaire for Sub-Saharan Africa. Chi-square tests were used to evaluate between-group differences in categorical variables pre- and post-intervention.

The continuous variables included knowledge scores for food type for an infant, exclusive breastfeeding, complementary feeding, and total knowledge scores. Student’s paired *t*-tests evaluated within-group differences in continuous variables. Between-group differences in mean knowledge scores were determined via Student’s independent *t*-test. Multivariate analyses were conducted (linear model) to determine the effects of use of the app on maternal IYCF knowledge. IBM SPSS Statistics for Windows, Version 29.0.2.0 was used to analyze all data [45].

## 4. Results

Table 1 presents the baseline sociodemographic characteristics of the 194 Nigerian teenage mother–child dyads in the control and intervention groups. Three-quarters or more of the control and intervention groups had attended secondary school (72.1%), were married (>87%) and lived with husbands (>73%). Over 40% of participants in both groups earned no income, and almost 60% of households earned 20,000 Nigerian Naira (USD 12) or less monthly. Over half of the children were boys, and almost half were less than six months of age. No significant differences were observed between both groups in all baseline socio-demographic characteristics (*p* < 0.01). Post-intervention, the proportion of teenage mothers earning no income declined to 16.5% in the control group and 26.8% in the BabyThrive group, while participants earning incomes up to 20,000 Nigerian Naira (USD 12) increased in both groups (77% vs. 68.0%, *p* = 0.185).

**Figure 2:**
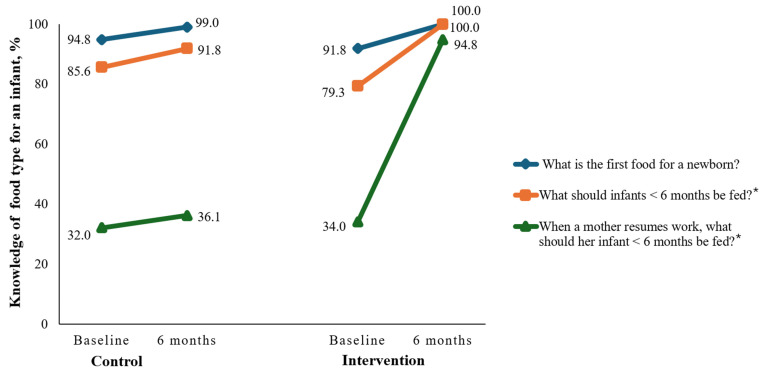
Baseline and post-intervention knowledge of Nigerian teenage mothers on food type for an infant. * *p* < 0.05.

Figure 2 presents baseline and post-intervention knowledge of Nigerian teenage mothers on food type for an infant in the control and intervention groups. At baseline, almost all participants in both groups (>90%) were cognizant that breast milk is the ideal first food for an infant. Similarly, four-fifths of both groups knew that children <6 months should be fed with breast milk. Nonetheless, only about one-third of participants in both groups were aware that expressed breast milk is the ideal option for an infant <6 months when the mother resumes work. The *p*-values on the chi-square test ranged from 0.389 to 0.902, indicating no significant differences between the control and intervention groups in baseline knowledge of food type for an infant. Post-intervention, the majority of the participants in both groups (>99%) chose breast milk only as the first food for a newborn (*p* > 0.05). Similarly, large proportions of respondents (>91%) selected breast milk only as the food type for an infant less than six months (*p* > 0.05). Post-intervention, only 36.1% of mothers in the control group, as compared to almost all intervention participants (94.8%), selected expressed breast milk as the ideal food for an infant when a mother resumes work (*p* < 0.05).

**Figure 3:**
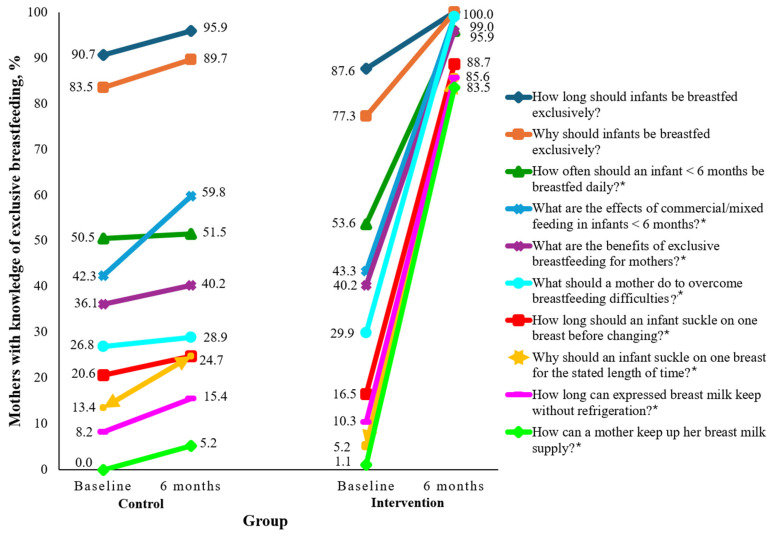
Baseline and post-intervention knowledge of Nigerian teenage mothers on breastfeeding. * *p* < 0.05.

Figure 3 presents the baseline and post-intervention knowledge of Nigerian teenage mothers on breastfeeding in the control and intervention groups. No differences in baseline knowledge of breastfeeding were observed between the control and BabyThrive groups, as Chi-square *p*-values ranged from 0.059 to 0.885. At baseline (>87%) and post-intervention (>95%), the majority of the mothers in both groups knew that infants should be breastfed exclusively for six months for optimum health and growth (*p* > 0.05). Similarly, large proportions of mothers (>75%) in both groups knew that infants should be breastfed exclusively for optimum growth, health, disease prevention and intelligence. Knowledge that breast milk supply can be maintained by breastfeeding more often was scant in both groups at baseline, as all but 1% of mothers believed breast milk supply can be maintained by drinking hot liquids, eating specific foods like cereal grains, or eating larger quantities of food. Post-intervention, there was an 82.4% increase in knowledge of how to maintain breast milk supply in the intervention group, as compared to 5.2% in the control group (*p* < 0.05). Similarly, in comparison with almost all users of the BabyThrive app, less than half (40.2%) of the control group were able to identify the benefits of exclusive breastfeeding for mothers (*p* < 0.05). Post-intervention, 85.6% of participants in the intervention group, as compared to only about 15% in the control group, knew that breast milk can be kept safely for up to eight hours without refrigeration (*p* < 0.05). Similarly, less than one-fourth in the control vs. four-fifths in the intervention group knew how long an infant should be suckled on one breast before changing to the other, or that suckling for up to 20 min before changing would provide sufficient hind milk for nutrients and satiety (*p* < 0.05).

**Figure 4:**
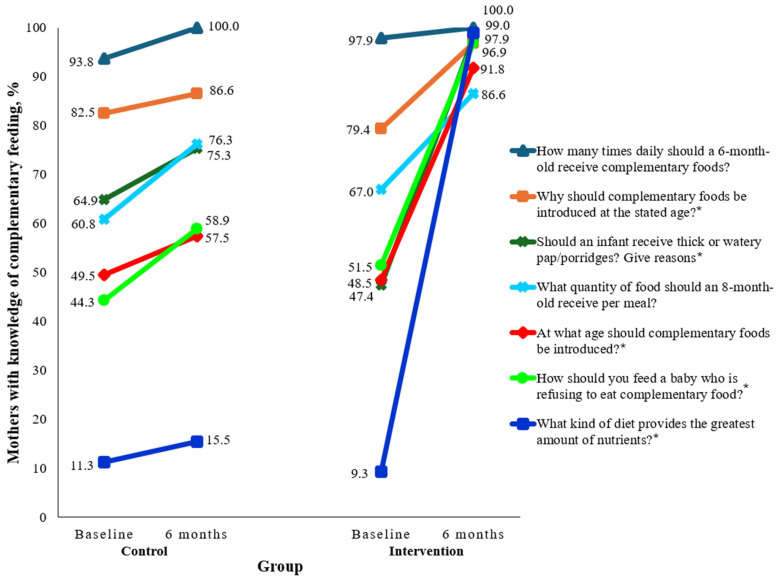
Baseline and post-intervention knowledge of complementary feeding of Nigerian teenage mothers. * *p* < 0.05.

Figure 4 presents the baseline and post-intervention knowledge of complementary feeding of Nigerian teenage mothers. At baseline, there were no significant differences in knowledge of complementary feeding between the control and intervention groups, as *p*-values on the Chi-square test ranged from 0.149 to 0.637. Knowledge of the minimum meal frequency was high in both groups, as almost all (>93%) at baseline and all participants post-intervention indicated that a six-month old child should receive at least two meals each day, in addition to breast milk. Post-intervention, <60% of mothers in the control group vs. >90% of the intervention group stated that complementary foods should be introduced at six months of age (*p* < 0.05). In contrast to >97% of BabyThrive users, <60% of the controls knew that responsive feeding was the best way to encourage a child to eat complementary food. Other participants in the control group reported not knowing what to do if a child refused complementary food or force-feeding as the best way to make the child eat. As compared to almost everyone in the intervention group, only one-sixth of mothers in the control group knew about dietary diversity, as reflected in the lack of familiarity with the four-star diet recommended by the World Health Organization (this diet contains a minimum of four food groups).

**Figure 5:**
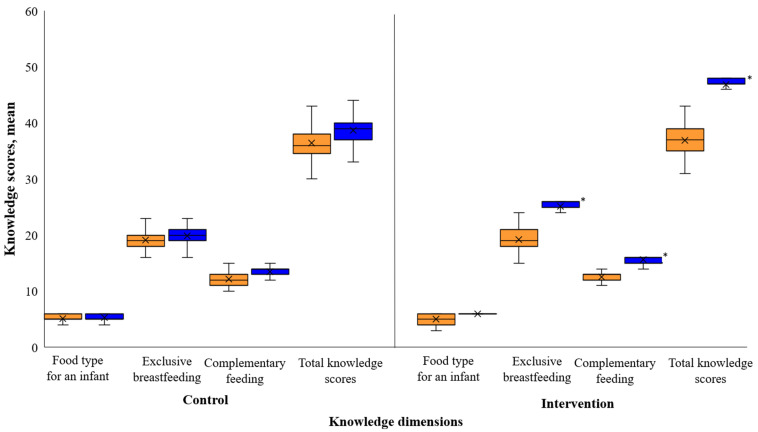
Mean knowledge scores of Nigerian teenage mothers at baseline and post-intervention. The orange color = baseline, blue = post-intervention. * *p* < 0.05.

Figure 5 presents the mean child feeding knowledge scores of Nigerian teenage mothers at baseline and post-intervention. Initially, the maternal knowledge scores did not differ significantly in any of the IYCF knowledge parameters (*p* > 0.05) between groups. Post-intervention, mean knowledge of food type for an infant (including ideal first food for a newborn, what to feed infants < 6 months, and what to feed an infant when mother returns to work) was slightly higher (5.97 ± 0.14) in the BabyThrive group as compared to the controls (5.27 ± 0.62, *p* > 0.05). Mean knowledge scores on exclusive breastfeeding (25.17 ± 1.37 vs. 19.87 ± 1.80), complementary feeding (15.68 ± 0.60, vs. 13.51 ± 1.21) and total knowledge scores (46.83 ± 1.79 vs. 38.65 ± 2.71) were substantially higher in the BabyThrive group as compared to the control group (*p* < 0.01).

In Table 2, a multivariate linear model of the effects of utilization of the BabyThrive app on maternal knowledge of infant and young child feeding is shown. The utilization of this app significantly increased knowledge of the food type for an infant, exclusive breastfeeding, complementary feeding, and total knowledge scores by 0.71–8.19 points, respectively (Hotelling’s T = 391.382, *p* = 0.000). A second multivariate linear model was adjusted for maternal education, personal income, employment status, and the interactions between BabyThrive participation, maternal education, and personal income, respectively. In this adjusted model, the significant effects of the BabyThrive app utilization on maternal knowledge persisted (Hotelling’s T = 94.311, *p* = 0.000). After taking account of these cofactors and covariates, the difference between groups in terms of knowledge of food type for an infant doubled to 1.58 points (*p* < 0.05). Although group differences remained significant in the adjusted model, slight reductions in the magnitude of the effects of the intervention were observed in knowledge of exclusive breastfeeding (reduced to 3.80 points), complementary feeding (declined to 1.97 points) and total knowledge scores (lowered to 7.36). The interactions between BabyThrive participation and maternal education significantly influenced the knowledge of breastfeeding (*p* = 0.015) and total knowledge scores (*p* = 0.008), as mothers in the control group who had not received any education earned lower scores in comparison with mothers who had received either primary, secondary or tertiary education.

## 5. Discussion

In this study, we conducted a randomized controlled trial to determine the effects of the BabyThrive app on maternal IYCF knowledge. The BabyThrive group had significantly improved maternal knowledge of food type for an infant, exclusive breastfeeding and complementary feeding, as well as total knowledge scores.

This investigation employed a parallel design, with a study duration of six months. Although a parallel design requires a large sample size, it is still the most commonly utilized in clinical trials [46]. This design has the advantage of the absence of carry-over effects, a shorter study duration and a reduced number of required visits [46]. In Senegal, an mHealth intervention (duration, 4 weeks) significantly improved complementary feeding practices in households with children 6–23 months [47]. In Pakistan, an mHealth intervention for 6 months improved IYCF knowledge, attitude and practices in pregnant women in their third trimester, and mothers with children 0–24 months [23]. In Iran, a mobile app-based IYCF intervention for 6 months increased weight-for-height z-scores by +0.65 in children 0–36 months of age [48]. Collectively, these studies illustrate that the study duration of 6 months is an effective period of time to improve malnutrition in very young children.

In the present research, no significant differences were observed between the BabyThrive (100%) and control (98.9%) groups in terms of knowledge of the ideal first food for an infant. This is similar to the findings of a cross-sectional investigation of maternal knowledge in rural Egypt, in which all participants knew that breast milk was the best source of nutrition for a newborn [49]. Nonetheless, the proportion of participants (100%) in rural Egypt who knew the benefits of breastfeeding for the mother was similar to that in the BabyThrive group, but greatly surpassed that of the control group in the present trial [49]. Post-intervention, wide disparities (94.8 vs. 36.1%) were observed between the BabyThrive and control groups in the current investigation, with respect to knowledge that expressed breast milk is the ideal food for an infant < 6 months if the mother is not at home. Note that this age is often when the mother resumes work. This finding is consistent with that of Talbert et al., who qualitatively explored knowledge of first-time mothers concerning breast milk expression in rural Kenya [50]. The findings of the present research of 95.9% in the intervention vs. 51.5% of controls having knowledge about on-demand breastfeeding (≥8–12 times daily) are consistent with those from a personalized breastfeeding counseling intervention in China [51]. In that study, knowledge of on-demand breastfeeding was significantly higher in the intervention group, as compared to the controls (95.1% vs. 68.1%) [51].

Perceived insufficient milk supply has been shown to be a strong determinant of early breastfeeding cessation [52]. A meta-analysis of 17 studies investigated the effectiveness of interventions in ameliorating perceived insufficient milk supply [53]. In that review, factors that promoted maternal breastfeeding self-efficacy included information about the importance of colostrum, the proper latching on of an infant to the breast, ways to deal with engorgement, the expression and storage of breast milk on return to work, the understanding of infant hunger cues, vicarious learning and social/verbal persuasion [53]. Ultimately, perceptions of the sufficiency of breast milk supply were improved [53]. In the current study, the BabyThrive app incorporated engaging, animated information on all of these concepts. The information provided is believed to explain the wide disparities observed between BabyThrive participants and controls, in terms of the knowledge of how to improve milk supply (83.5 vs. 5.2%), and how to deal with breastfeeding difficulties (99.0% vs. 28.9%).

It should be noted that due to the absence of sophisticated cooling technology and erratic power supply, an awareness of the duration of safety of expressed breast milk without refrigeration can promote breastfeeding exclusivity in low-income settings. Also, findings from studies in rural communities in African countries show that the practice of expressing breast milk is regarded as culturally unacceptable [50,54]. In a cross-sectional study in rural Kenya that examined knowledge of breast milk expression, 66% of working mothers did not have adequate knowledge of breast milk expression and storage [55]. This is in agreement with findings among the control group in our study, that only 19% of mothers exhibited knowledge about how long expressed breast milk can be kept without refrigeration. However, the findings in Kenya are in stark contrast with the results of the present intervention, in which 88.3% of BabyThrive participants had adequate knowledge of the duration of safety of expressed breast milk without refrigeration [55].

Hind milk (the milk that is released towards the end of a breastfeeding or breast milk expression session) has been shown to contain 2–3 times the amount of fat found in fore milk (milk at the start of a feed or expression session) [56,57]. However, a previous study concluded that there is no significant difference in the fat contents of fore and hind milk [58]. In contrast, a recent chemical evaluation of human milk reported consistently higher fat contents in hind milk, and significantly greater amounts of human milk oligosaccharides in hind milk, as compared to fore milk [59]. A recent prospective investigation that fed hind milk to preterm infants produced significant increments in weight (mean difference of 3.9 g/kg/day), weight z-scores (mean difference of 0.61) and head circumference z-scores (mean difference of 0.83), as compared to energy- and protein-optimized, full, enteral feeds [56]. Thus, knowledge of the importance of hind milk can contribute to the success of exclusive breastfeeding, as this promotes awareness of its contribution to infant satiety and growth. A breastfeeding intervention that promoted exclusive breastfeeding via peer supporters among mothers of hospitalized, malnourished children in rural Kenya improved the knowledge of the importance of hind milk in half of the study population [55]. The findings of the present investigation are in agreement with this peer support intervention; a 60.9% discrepancy in knowledge of the benefits of hind milk was observed between participants who were exposed and those non-exposed to the BabyThrive intervention [55].

The inappropriate timing of the introduction of complementary foods (too early or too late) can result in negative effects on child nutrition, growth and health [60,61]. In the present research, only 57.5% of participants in the control group were aware of the appropriate time to introduce complementary foods, as compared to more than 90% of the BabyThrive group. The proportion of control participants with this knowledge in this study is substantially lower than the value of 77.7% reported in a cross-sectional investigation in Nepal [61].

Dietary diversity is crucial for optimum child nutrition, growth and health. An mHealth intervention in Nepal improved maternal IYCF knowledge scores and child dietary diversity 1.36-fold [62]. These results are in agreement with the findings of the present research. In the current investigation, wide disparities were observed between the BabyThrive app users (99%) and control (15.5%) groups in terms of the knowledge of the four-star diet, an indicator of knowledge of dietary diversity [63]. In the current RCT, almost half (41.1%) of the participants in the control group did not know that responsive feeding is the best way to feed a fussy child refusing to eat complementary foods. The acceptance of the practice of force-feeding, the utilization of psychological or physical force to induce children to eat, was common in the control group. Consistent with the findings among control group participants in the present study, a cross-sectional investigation in eastern Nigeria revealed that only 46% of participants were aware of the adverse effects of force-feeding [64]. Nonetheless, almost all participants in the BabyThrive group (96.8%) knew about responsive feeding.

A recent meta-analysis that included 18 breastfeeding studies showed inconclusive results, with respect to the effects of mHealth interventions on IYCF [65]. However, several recent mHealth interventions have shown significant improvements in maternal IYCF knowledge [23,66,67]. For example, a 14.8% increase in the proportion of mothers with overall IYCF knowledge was reported in an mHealth intervention delivered via text messages in rural Pakistan [23]. In Indonesia, an mHealth intervention resulted in a 1.45-fold increase in overall child feeding knowledge [66]. In India, an mHealth app deployed to improve maternal knowledge concerning preterm care elevated maternal IYCF knowledge by 10 points, as compared to 2 points in controls [67]. In Australia, use of the My Baby Now app produced a 6.9% increase in breastfeeding knowledge [68]. Collectively, these results are comparable to the findings of the present investigation, in which mean knowledge scores were significantly higher in the BabyThrive group. Today, mobile serious games are increasingly being utilized for nutrition education, due to their engaging and entertaining nature and capacity to modify behavior [32,33,34]. Nonetheless, few evidence-based games for improving maternal knowledge exist. Similar to the findings of this study, which produced significant improvements in knowledge after BabyThrive utilization, a mobile game called Foodbot Factory deployed in Canada significantly improved the overall nutrition knowledge of children by 3.2 points over the control group [69].

The effects of interventions aimed at increasing knowledge can be modulated by personal factors and covariates, including pre-intervention knowledge, educational attainment and other socio-demographic characteristics. The “knowledge gap hypothesis” postulates that individuals in higher sociodemographic strata are predisposed to acquiring knowledge more rapidly than those at a lower stratum [70]. Previous research has shown relationships between the educational status of mothers, occupation, income, other covariates and knowledge concerning childcare [71,72,73]. Thus, it is pertinent to adjust for the effects of covariates and factors extraneous to the intervention that may potentially confound the outcomes observed. The BabyThrive app intervention significantly improved the four dimensions of IYCF knowledge investigated. These significant effects persisted after adjusting for sociodemographic factors. These factors included maternal educational attainment and income, and the interactions between app utilization, maternal education, and personal income, respectively (Hotelling’s T = 391.382, *p* = 0.000). Nonetheless, the interaction between BabyThrive participation and maternal education was significant, as mothers in the control group who had no education exhibited lower scores in terms of knowledge of breastfeeding and total knowledge scores, in comparison with their educated counterparts.

The findings of the present research are generalizable to developing countries and other low-income countries. One limitation of this study is the absence of extensive game usage data due to the offline nature of the app. Although the game includes a button for users to upload data, low mobile phone storage space, poor access and affordability of internet connectivity, and phone damages/losses made it difficult for users to upload data. Another limitation was the need to reinstall the app multiple times for some participants due to lost/stolen or damaged devices.

## 6. Conclusions

This research evaluated the impact of BabyThrive, a mobile gaming app, on the knowledge of infant and young child feeding of Nigerian teenage mothers. This app significantly increased maternal IYCF knowledge in comparison with controls. Thus, the BabyThrive app is a useful instrument for changing the IYCF knowledge of mothers. It is ideal for nutritionists and other health personnel, governments, intergovernmental agencies, international non-profits, and other stakeholders involved in the dissemination of IYCF information to mothers. Future studies should investigate the scalability of the app to other contexts across the globe.

## Figures and Tables

**Table 1:** Baseline sociodemographic characteristics of Nigerian teenage mother–child dyads.

Baseline Characteristics	Control		Intervention		*p*-Value
Mother	N	%	N	%	
Age, years					0.500
15–17	19	19.6	20	20.6	
18–19	78	80.4	77	79.4	
Education					0.836
None	10	10.3	7	7.2	
Primary	15	15.5	17	17.5	
Secondary	70	72.1	70	72.1	
Tertiary	2	2.1	3	3.2	
Marital status					0.138
Single, separated, cohabiting	6	6.2	12	12.4	
Married	91	93.8	85	87.6	
Living with					0.109
Husband	80	82.5	71	73.2	
Parents	14	14.4	16	16.5	
Other relatives	3	3.1	10	10.3	
Personal monthly income (Naira, USD)					0.826
None	41	42.3	42	43.3	
≤20,000 Naira (≤USD 12)	55	56.7	53	54.6	
>20,000 Naira (>USD 12)	1	1.0	2	2.1	
Household monthly income (Naira, USD)					0.573
Not known	24	24.7	20	20.6	
≤20,000 (≤USD 12)	33	34.0	40	41.2	
≥20,000 (≥USD 12)	40	41.2	37	38.1	
Child					
Sex					0.248
Male	50	51.5	58	59.8	
Female	47	48.5	39	40.2	
Age, months					
0–5	41	42.3	48	49.5	0.087
6–11	29	29.9	36	37.1	
12–17	23	23.7	12	12.4	
18–23	4	4.1	1	1.0	

**Table 2:** Multivariate linear model of the effects of the utilization of BabyThrive on maternal knowledge of child feeding.

Child Feeding Knowledge	β Estimate ^a^	Standard Errors	Student’s *t* ^b^	95% Confidence Intervals	*p*
Food type for an infant					
Control ^c^	-	-	-	-	-
Intervention	0.71	0.07	10.99	0.58–0.84	0.000 *
Exclusive breastfeeding					
Control	-	-	-	-	-
Intervention	5.31	0.23	23.07	4.86–5.76	0.000 *
Complementary feeding					
Control	-	-	-	-	-
Intervention	2.17	0.14	15.78	1.89–2.44	0.000 *
Total knowledge score					
Control	-	-	-	-	-
Intervention	8.19	0.33	24.81	7.54–8.84	0.000 *

^a^ β estimate = regression coefficient. ^b^ Student’s *t* = test statistic. ^c^ Control = reference category. * *p* < 0.05.

## Data Availability

Data are available upon reasonable request from the authors.

## References

[1] Government of the Federal Republic of Nigeria, United Nations Children’s Fund (2023). Formative Research on Key Child Survival and Nutrition Practices in the First 1000 Days of Life: Findings.

[2] Nigeria Multidimensional Poverty Index 2022 National Bureau of Statistics. https://www.nigerianstat.gov.ng/pdfuploads/NIGERIA%20MULTIDIMENSIONAL%20POVERTY%20INDEX%20SURVEY%20RESULTS%202022.pdf.

[3] (2021). Indicators for Assessing Infant and Young Child Feeding Practices: Definitions and Measurement Methods.

[4] Walters D.D., Phan L.T.H., Mathisen R. (2019). The cost of not breastfeeding: Global results from a new tool. Health Policy Plan..

[5] Jovita N., Eustace E. (2024). Teenage Pregnancy in Nigeria from 1970 to 2023: Burden, Issues, and Prospects. Glob. J. Med. Clin. Case Rep..

[6] National Population Commission (NPC) [Nigeria], ICF (2019). Nigeria Demographic and Health Survey 2018.

[7] UNICEF (2013). The Community Infant and Young Child Feeding Counselling Package. https://www.unicef.org/documents/community-iycf-package.

[8] Ameh N., Oyefabi A.M., Hawwa M.N. (2022). Marriage to first pregnancy interval and related factors among women in North Central Nigeria. Ann. Afr. Med..

[9] Hoddinott J., Karachiwalla N.I., Ledlie N.A., Roy S. (2016). Adolescent girls’ infant and young child nutrition knowledge levels and sources differ. Matern. Child Nutr..

[10] Salami K.K., Ayegboyin M., Adedeji I.A. (2014). Unmet social needs and teenage pregnancy in Ogbomosho, South-western Nigeria. Afr. Health Sci..

[11] Shiffman J., Kunnuji M., Shawar Y.R., Robinson R.S. (2018). International norms and the politics of sexuality education in Nigeria. Glob. Health.

[12] Eno-Abasi Sunday Removal of Sex Education from Curriculum: Is Nigeria Scoring Another Own Goal? The Guardian Newspaper. https://guardian.ng/features/removal-of-sex-education-from-curriculum-is-nigeria-scoring-another-own-goal/#:~:text=On%20Thursday%2C%20November%203%2C%20the,Basic%20Education%20Curriculum%20(BEC).

[13] Envuladu E.A., Issaka A.I., Dhami M.V., Sahiledengle B., Agho K.E. (2023). Differential Associated Factors for Inadequate Receipt of Components and Non-Use of Antenatal Care Services among Adolescent, Young, and Older Women in Nigeria. Int. J. Environ. Res. Public Health.

[14] Ayuba I.I., Gani O. (2012). Outcome of teenage pregnancy in the Niger delta of Nigeria. Ethiop. J. Health Sci..

[15] Mekonnen T., Dune T., Perz J. (2019). Maternal health service utilisation of adolescent women in sub-Saharan Africa: A systematic scoping review. BMC Pregnancy Childbirth.

[16] Olutade-Babatunde O., van Der Kwaak A., Bet-ini N.C., Keshinro M.I. (2024). A Critical Review of Factors Affecting Health-Seeking Behavior Among Adolescent Mothers in Nigeria: Towards Inclusive and Targeted Interventions. medtigo J. Med..

[17] Adeyemi O., Afolabi W.A., Ferguson E., Hoyt J., Lawal H., Okunola R.A., Webster J., Yohanna-Dzingina C. How to Strengthen the Infant and Young Child Feeding (IYCF) Programme in Northern Nigeria. https://opendocs.ids.ac.uk/opendocs/handle/20.500.12413/6906.

[18] De Buhr E., Tannen A. (2020). Parental health literacy and health knowledge, behaviours and outcomes in children: A cross-sectional survey. BMC Public Health.

[19] Arikpo D., Edet E.S., Chibuzor M.T., Odey F., Caldwell D.M. (2018). Educational interventions for improving primary caregiver complementary feeding practices for children aged 24 months and under. Cochrane Database Syst. Rev..

[20] Elsahoryi N., Altamimi E., Subih H.S., Hammad F.J., Woodside J.V. (2020). Educational intervention improved parental knowledge, attitudes, and practices (KAP) and adherence of patients with celiac disease to gluten-free diet. Int. J. Food Sci..

[21] Aboagye R.G., Seidu A.A., Ahinkorah B.O., Cadri A., Frimpong J.B., Dadzie L.K., Budu E., Eyawo O., Yaya S. (2023). Prevalence and predictors of infant and young child feeding practices in sub-Saharan Africa. Int. Health.

[22] Setia A., Shagti I., Boroa R.M., Adi A.M., Saleh A., Amryta P. (2020). The effect of family-based nutrition education on the intention of changes in knowledge, attitude, behavior of pregnant women and mothers with toddlers in preventing stunting in Puskesmas Batakte. People.

[23] Akber S., Mahmood H., Fatima R. (2019). Effectiveness of a mobile health intervention on infant and young child feeding among children ≤ 24 months of age in rural Islamabad over six months duration. F1000Research.

[24] World Health Organization (2011). mHealth: New Horizons for Health Through Mobile Technologies.

[25] Beres D. (2018). Africa Phone Study. The Huffington Post.

[26] Pew Research Center (2018). Internet Connectivity Seen As Having Positive Impact on Life in Sub-Saharan Africa. https://www.pewresearch.org/global/wp-content/uploads/sites/2/2018/10/Pew-Research-Center_Technology-use-in-Sub-Saharan-Africa_2018-10-09.pdf.

[27] Doris Dokua Sasu Population of Nigeria 1950–2024. Statista. https://www.statista.com/statistics/1122838/population-of-nigeria.

[28] Pete Bell Nigeria: Africa’s Mobile Giant. Telegeography. https://blog.telegeography.com/nigeria-africas-mobile-giant#:~:text=Nigeria%20is%20home%20to%20Africa’s,209.5%20million%20a%20year%20earlier.

[29] Akinfaderin-Agarau F., Chirtau M., Ekponimo S., Power S. (2012). Opportunities and limitations for using new media and mobile phones to expand access to sexual and reproductive health information and services for adolescent girls and young women in six Nigerian states. Afr. J. Reprod. Health.

[30] Chen H., Chai Y., Dong L., Niu W., Zhang P. (2018). Effectiveness and appropriateness of mHealth interventions for maternal and child health: Systematic review. JMIR Mhealth Uhealth.

[31] Kazemi D.M., Borsari B., Levine M.J., Lamberson K.A., Dooley B. (2018). REMIT: Development of a mHealth theory-based intervention to decrease heavy episodic drinking among college students. Addict. Res. Theory.

[32] Pereira C.V., Figueiredo G., Esteves M.G., de Souza J.M. We4Fit: A game with a purpose for behavior change. Proceedings of the 2014 IEEE 18th International Conference on Computer Supported Cooperative Work in Design.

[33] Thompson D. (2012). Designing serious video games for health behavior change: Current status and future directions. J. Diabetes Sci. Technol..

[34] Lamas S., Rebelo S., da Costa S., Sousa H., Zagalo N., Pinto E. (2023). The influence of serious games in the promotion of healthy diet and physical activity health: A systematic review. Nutrients.

[35] Hammady R., Arnab S. (2022). Serious Gaming for Behaviour Change: A Systematic Review. Information.

[36] Abt C.C. (1970). Serious Games.

[37] Zyda M. (2005). From visual simulation to virtual reality to games. Comput. Educ..

[38] Andrew L., Barwood D., Boston J., Masek M., Bloomfield L., Devine A. (2023). Serious games for health promotion in adolescents—A systematic scoping review. Educ. Inf. Technol..

[39] Katara A., Verma M., AP K. (2024). The Effect of Video Game on Behavior of Adolescents. Am. J. Nurs..

[40] Alghamdi A.S., Bitar H.H. (2023). The positive impact of gamification in imparting nutritional knowledge and combating childhood obesity: A systematic review on the recent solutions. Digit. Health.

[41] Sari A.S., Aprianti N.F. (2023). The effect of simulation puzzle games on increasing mother’s knowledge about stunting prevention and feeding patterns in children aged 0–24 Months. Babali Nurs. Res..

[42] Sosanya M.E., Samuel F.O., Bashir S., Omoera V.O., Freeland-Graves J.H. (2024). A mobile gaming app to train teenage mothers on appropriate child feeding practices: Development and validation study. J. Med. Internet Res..

[43] Faul F., Erdfelder E., Lang A.G., Buchner A. (2007). G*Power 3: A flexible statistical power analysis program for the social, behavioral, and biomedical sciences. Behav. Res. Methods.

[44] Sosanya M.E., Beamon I., Muhammad R., Freeland-Graves J.H. (2023). Development and validation of the teen moms child feeding Questionnaire for Sub-Saharan Africa. BMC Public Health.

[45] IBM Corp (2023). IBM SPSS Statistics for Windows, Version 29.0.2.0.

[46] Kim S. (2024). Overview of clinical study designs. Clin. Exp. Emerg. Med..

[47] Downs S.M., Sackey J., Kalaj J., Smith S., Fanzo J. (2019). An mHealth voice messaging intervention to improve infant and young child feeding practices in Senegal. Matern. Child Nutr..

[48] Seyyedi N., Rahimi B., Eslamlou H.R.F., Afshar H.L., Spreco A., Timpka T. (2020). Smartphone-based maternal education for the complementary feeding of undernourished children under 3 years of age in food-secure communities: Randomised controlled trial in Urmia, Iran. Nutrients.

[49] Mohammed E.S., Ghazawy E.R., Hassan E.E. (2014). Knowledge, attitude, and practices of breastfeeding and weaning among mothers of children up to 2 years old in a rural area in El-minia governorate, Egypt. J. Fam. Med. Prim. Care.

[50] Talbert A.W., Tsofa B., Mumbo E., Berkley J.A., Mwangome M. (2018). Knowledge of, and attitudes to giving expressed breastmilk to infants in rural coastal Kenya; focus group discussions of first time mothers and their advisers. Int. Breastfeed. J..

[51] Huang P., Yao J., Liu X., Luo B. (2019). Individualized intervention to improve rates of exclusive breastfeeding: A randomized controlled trial. Medicine.

[52] De Roza J.G., Fong M.K., Ang B.L., Sadon R.B., Koh E.Y., Teo S.S. (2019). Exclusive breastfeeding, breastfeeding self-efficacy and perception of milk supply among mothers in Singapore: A longitudinal study. Midwifery.

[53] Galipeau R., Baillot A., Trottier A., Lemire L. (2018). Effectiveness of interventions on breastfeeding self-efficacy and perceived insufficient milk supply: A systematic review and meta-analysis. Matern. Child Nutr..

[54] Edemba P.W., Irimu G., Musoke R. (2022). Knowledge attitudes and practice of breastmilk expression and storage among working mothers with infants under six months of age in Kenya. Int. Breastfeed. J..

[55] Kahindi J., Jones C., Berkley J.A., Mwangome M. (2020). Establishing exclusive breastfeeding among in-patient malnourished infants in a rural Kenyan hospital: Mothers’ experiences of a peer supporter intervention. Int. Breastfeed. J..

[56] Alshaikh B.N., Festival J., Reyes Loredo A., Yusuf K., Towage Z., Fenton T.R., Wood C. (2023). Hindmilk as a rescue therapy in very preterm infants with suboptimal growth velocity. Nutrients.

[57] Ballard O., Morrow A.L. (2013). Human milk composition: Nutrients and bioactive factors. Pediatr. Clin. N. Am..

[58] Michalski M.C., Briard V., Michel F., Tasson F., Poulain P. (2005). Size distribution of fat globules in human colostrum, breast milk, and infant formula. J. Dairy Sci..

[59] Pu Z., Sun Z., Liu J., Zhang J., Cheng M., Zhang L., Zhou P. (2023). Differences in the content of human milk oligosaccharides between foremilk and hind milk. Food Bioeng..

[60] Srivastava S., Chaturvedi N. (2020). Complementary feeding practices: A critical intervention for survival and well-being of children. Int. J. Recent Sci. Res..

[61] Basnet S., Sathian B., Malla K., Koirala D.P. (2015). Reasons for early or late initiation of complementary feeding: A study in Pokhara. Am. J. Public Health Res..

[62] Cunningham K., Cech S., Gupta A.S., Rana P.P., Humphries D., Frongillo E.A. (2024). Text messages to improve young child diets: Results from a cluster-randomized controlled trial in Kanchanpur, Nepal. Matern. Child Nutr..

[63] Thiha K., Cho T.A., Lwin A. (2022). Are socio-economic characteristics and maternal dietary patterns dominant factors on four-star diet achievement of infants and young children (6–23 months)?. Int. J. Community Med. Public Health.

[64] Ndu I., Ekwochi U., Osuorah C., Chinawa J., Asinobi I., Eze J., Amadi O., Egwuonwu A. (2016). The knowledge and practice of forced-feeding among mothers and caregivers in Enugu, South East Nigeria. Int. J. Trop. Dis. Health.

[65] Knop M.R., Nagashima-Hayashi M., Lin R., Saing C.H., Ung M., Oy S., Yam E.L.Y., Zahari M., Yi S. (2024). Impact of mHealth interventions on maternal, newborn, and child health from conception to 24 months postpartum in low- and middle-income countries: A systematic review. BMC Med..

[66] Siswati T.S., Sitasari A., Paramashanti B.A., Tjaronosari T., Nurhidayat N., Wijanarka A., Waris L. (2024). Effect of mHealth based intervention on maternal knowledge and practices of child care: A quasi-experimental study. Public Health Indones..

[67] Phagdol T., Nayak B.S., Lewis L.E., Bhat R., Guddattu V. (2023). Effectiveness of mHealth application in improving knowledge of mothers on preterm home care. J. Neonatal Nurs..

[68] Laws R.A., Denney-Wilson E.A., Taki S., Russell C.G., Zheng M., Litterbach E.K., Ong K.L., Lymer S.J., Elliott R., Campbell K.J. (2018). Key lessons and impact of the growing healthy mhealth program on milk feeding, timing of introduction of solids, and infant growth: Quasi-experimental study. JMIR Mhealth Uhealth.

[69] Froome H.M., Townson C., Rhodes S., Franco-Arellano B., LeSage A., Savaglio R., Brown J.M., Hughes J., Kapralos B., Arcand J. (2020). The effectiveness of the foodbot factory mobile serious game on increasing nutrition knowledge in children. Nutrients.

[70] Tichenor P.J., Donohue G.A., Olien C.N. (1970). Mass media flow and differential growth in knowledge. Public Opin. Q..

[71] Mohini H., Sumanth Shetty B. (2017). A study to assess the knowledge of mothers on home based neonatal care at selected area of rural Bangalore. Int. J. Community Med. Public Health.

[72] Bornstein M.H., Cote L.R., Haynes O.M., Hahn C.S., Park Y. (2010). Parenting knowledge: Experiential and sociodemographic factors in European American mothers of young children. Dev. Psychol..

[73] Obonyo K.O., Kaindi D.W.M., Ngala S., Kogi-Makau W. (2024). Maternal nutrition knowledge and mothers’ ability to utilize mobile phone application for health information sharing at Kenyatta National Hospital, Nairobi city, Kenya. Afr. J. Food Agric. Nutr. Dev..

